# A Highly Potent SARS-CoV-2 Blocking Lectin Protein

**DOI:** 10.1021/acsinfecdis.2c00006

**Published:** 2022-04-15

**Authors:** Recep E. Ahan, Alireza Hanifehnezhad, Ebru Ş. Kehribar, Tuba C. Oguzoglu, Katalin Földes, Cemile E. Özçelik, Nazlican Filazi, Sıdıka Öztop, Fahreddin Palaz, Sevgen Önder, Eray U. Bozkurt, Koray Ergünay, Aykut Özkul, Urartu Özgür Şafak Şeker

**Affiliations:** † UNAM-Institute of Materials Science and Nanotechnology, Bilkent University, Ankara 06800, Turkey; ‡ Faculty of Veterinary Medicine, Department of Virology, Ankara University, Ankara 06110, Turkey; § Adana Dr. Turgut Noyan Medical and Research Center, Department of Immunology, Baskent University, Adana 01250, Turkey; ∥ Faculty of Medicine, Department of Medical Pathology, Hacettepe University, Ankara 06230, Turkey; ⊥ Faculty of Medicine, Department of Medical Microbiology, Virology Unit, Hacettepe University, Ankara 06230, Turkey; # Biotechnology Institute, Ankara University, Ankara 06135, Turkey; □ Faculty of Medicine, Hacettepe University, Ankara 06230, Turkey

**Keywords:** Please add keywords

## Abstract

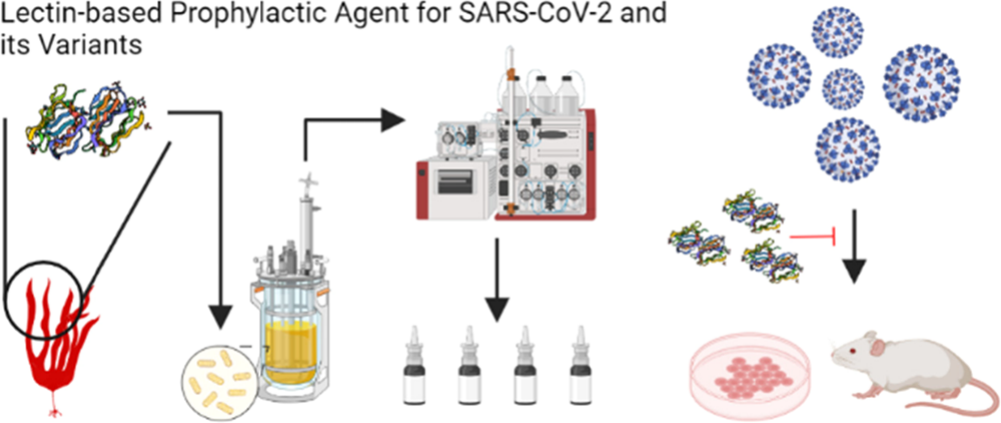

The COVID-19 (coronavirus disease-19) pandemic affected more than 180 million people around the globe, causing more than five million deaths as of January 2022. SARS-CoV-2 (severe acute respiratory syndrome coronavirus 2), the new coronavirus, has been identified as the primary cause of the infection. The number of vaccinated people is increasing; however, prophylactic drugs are highly demanded to ensure secure social contact. A number of drug molecules have been repurposed to fight against SARS-CoV-2, and some of them have been proven to be effective in preventing hospitalization or ICU admissions. Here, we demonstrated griffithsin (GRFT), a lectin protein, to block the entry of SARS-CoV-2 and its variants, Delta and Omicron, into the Vero E6 cell lines and IFNAR^–/–^ mouse models by attaching to the spike protein of SARS-CoV-2. Given the current mutation frequency of SARS-CoV-2, we believe that GRFT protein-based drugs will have a high impact in preventing the transmission of both the Wuhan strain as well as any other emerging variants, including Delta and Omicron variants, causing the high-speed spread of COVID-19.

SARS-CoV-2 (severe acute respiratory syndrome coronavirus 2) is the causative agent of COVID-19 (coronavirus disease-19), which has become a pandemic and a global health threat since its emergence in December 2019.^1^ Air-borne human-to-human transmission of SARS-CoV-2 caused the spread of the virus to almost all countries in less than a year.^2^ Due to the high transmission rate, mandatory face mask use and social distancing are implemented in many countries; meanwhile, massive nucleic acid testing is used to suppress the further spread via finding and isolating asymptomatic and presymptomatic patients.^3^

The SARS-CoV-2 genome is encoded in a positive-single-stranded RNA molecule that has six protein-coding frames consisting of ORF1ab, spike glycoprotein (S), nucleocapsid (N), membrane (M), and envelope (E) proteins.^4^ S protein, which shares moderate similarity with SARS-CoV, contains a receptor-binding domain (RBD) that binds the ACE2 protein as the cell surface receptor to initiate viral invasion.^5^ S protein is found as a trimer with two metastable conformations in which the RBD is either in the “up” or “down” state. Upon binding to ACE2, S1 and S2 domains of S are cleaved by surface proteases such as TMPRSS2 or cathepsin L. Cleavage of S protein domains causes irreversible structural changes and primes for the viral fusion.^6^ Due to high antigenicity of S proteins and importance of ACE2 binding for virus entrance, many prophylactic vaccines based on mRNA (BioNTech/Pfizer^7^ and Moderna^8^), adenoviral vectors (J&J^9^ and AstraZeneca/Oxford^10^), and recombinant antigens (Novavax^11^) utilize S proteins to induce anti-SARS-CoV-2 immune response.

Aside from vaccines, current treatment options of COVID-19 include disruption of viral amplification by either inhibiting viral entrance to cells or the viral replication machinery. Drug repurposing studies successfully identified several small molecules such as remdesivir as a viral replication inhibitor that acts on RNA polymerase of SARS-CoV-2, which is also approved by the FDA for clinical use.^12^ Moreover, neutralizing antibody (nAb) therapies covering convalescent plasma transfer and recombinant monoclonal anti-spike antibodies are also approved for emergency use.^13,14^ Generally, nAbs hamper the binding of the RBD to ACE2 protein, thereby preventing virus internalization. Yet, some nAbs bind to regions other than the RBD in S protein to hinder conformational changes for the fusion state.^15^ Despite the apparent beneficial outcomes of nAb therapies among COVID-19 patients, limited availability of convalescent plasma and the costly production process of monoclonal antibodies restrain access to this treatment.^16^ In addition, the ongoing evolution of SARS-CoV-2 poses a significant risk, as mutation accumulation in S protein can lead to immune escape, which can cause reinfections and make nAb therapies as well as vaccination ineffective.

Many SARS-CoV-2 variants emerged, dominated, and were replaced by other variants during the course of COVID-19 pandemic.^17^ The Alpha/B.1.1.7 variant was the dominant strain due to its high transmissibility in the beginning of 2021 until the Delta/B1.617.2 variant emerged with superior antibody resistance compared to the Alpha strain.^18^ At the time this article is written, Omicron/B1.1.529 is the dominant viral strain among the people who get infected with SARS-CoV-2. A recent study indicated that high mutation accumulation in the RBD with at least 15 amino acids based on the sequencing data of the omicron strain enables the virus to escape the immune response acquired not only with natural infection but also with four different vaccines that are widely administered around the world.^19^ Full evolution potential of S protein still needs to be determined, yet it is highly likely that SARS-CoV-2 will acquire more mutations to cope with the pressure due to vaccine-elicited immunity, because the uneven distribution of vaccines provides viable hosts for evolution.^20^ Hence, new therapeutic agents that have distinct inhibitory action are required until complete eradication of SARS-CoV-2.

Lectin proteins isolated from seaweeds are shown to be potent antiviral agents against enveloped viruses, e.g., HIV-1,^21^ herpes virus,^22^ and two deadly human coronaviruses, SARS-CoV^23^ and MERS-CoV.^24^ Antiviral activity of seaweed lectins arises from their affinity to surface glycoproteins on viruses such as gp-120 protein of HIV-1 and spike proteins of SARS-CoV and MERS-CoV. Upon binding to surface proteins, lectins generally block the viral internalization step and thereby prevent the viral infection. In this study, we evaluated the activity of griffithsin lectin protein (GRFT) from *Griffithsia sp*. against the novel human coronavirus, SARS-CoV-2. For this purpose, GRFT is recombinantly expressed in *Escherichia coli* (*E. coli*) with a histidine tag and purified. Binding of recombinant GRFT to whole inactivated SARS-CoV-2 and purified spike protein from HEK293 are validated and characterized with enzyme-linked immunosorbent assay (ELISA), isothermal titration calorimetry (ITC), and quartz crystal microbalance (QCM). The activity of GRFT is assessed in vitro with Vero E6 cells and in vivo with IFNAR^–/–^ mice. Our results indicate that GRFT is a potent nonmutagenic antiviral agent against SARS-CoV-2, reducing virus transmission through blocking its entry into the cells. Although the vaccination of the world population is in progress, the time to reach herd immunity is still unknown. Also, recent mutation reports on different subunits of SARS-CoV-2 may urge the need for changing the current vaccine designs. In this regard, our proposed antiviral GRFT can help to suppress the transmission of the virus. Prevention of person-to-person transmission may also help to stop the evolutionary change of the virus through selective pressure. Upon very promising results from in vitro and in vivo assays and experiments, GRFT is formulated as a nasal spray for upcoming human phase trials. We believe that GRFT-based nasal spray will have the potential to change the current scenario of the pandemic.

## Results

### rGRFT Binds SARS-CoV-2 through rGRFT

**Figure 1:**
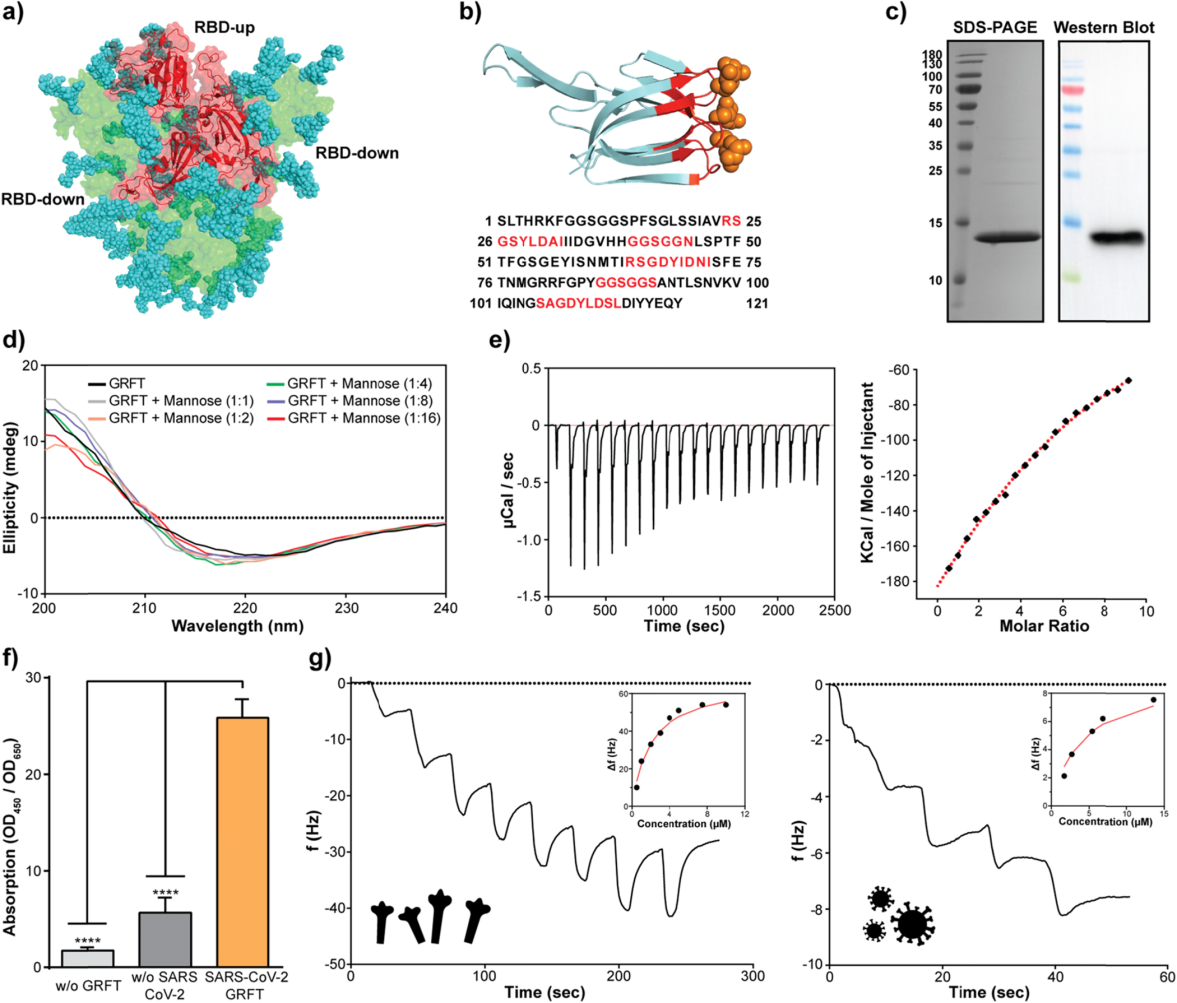
Binding kinetics of rGRFT to S protein of SARS-CoV-2 and the virus itself. (a) Structure of HEK293 produced recombinant SARS-CoV-2 spike protein, and the RBD with attached glycan structures colored blue (side view).^26^ (b) Structure and amino acid sequence of rGRFT. (c) Purification validation of rGRFT protein on SDS-PAGE and western blotting gels, single bands represent the expected 14.5 kDA molecular mass of GRFT. (d) Secondary structure changes of rGRFT upon titration with mannose. (e) Molecular binding interaction of SARS-CoV-2 spike protein with rGRFT protein analyzed with ITC. (f) Qualitative ELISA for the interaction of rGRFT protein with SARS-CoV-2 virus. (g) QCM-D-based quantitative analysis of the binding of rGRFT on purified spike (left) and inactivated SARS-CoV-2 (right) immobilized on a carboxylated gold surface.

Spike glycoprotein is heavily glycosylated with oligomannose and complex type sugars on its estimated 22 N-linked and 2 O-linked potential glycosylation sites per protomer. Total glycan structures, when recombinantly expressed in HEK293, account for the one-third molecular weight of spike protein and cover approximately 40% of the protein surface^25,26^ (Figure 1a). Owing to the high conservation of sequons among SARS-CoV and SARS-CoV-2 spike proteins,^27^ we hypothesized that GRFT protein can act as an antiviral for SARS-CoV-2, due to its previously reported antiviral effects on SARS-CoV. GRFT is considered as a domain-swapped dimer folded as a beta prism, which can bind three sugar molecules per protomer^28^ (Figure 1b).

To test our hypothesis, recombinant GRFT (rGRFT) was expressed with a 6× His-tag in *E. coli* BL21 (DE3) and was purified with a Ni-NTA column. The final yield of pure rGRFT was found to be approximately 12 mg/L consistent with previous reports at the shake-flask scale in LB media.^29^ Purity of rGRFT was calculated to be >95% via SDS-PAGE under nonreducing conditions. rGRFT purification was further validated with western blot using antibodies against His-tag (Figure 1c). The secondary structures of rGRFT were measured with circular dichroism (CD) both in the apo form and in complex with mannose sugar. In the CD spectrum, apo-rGRFT gave a minima around 218 nm, in agreement with its beta-sheet-rich 3D structure. Overall secondary structure elements were not changed upon addition of mannose up to 16-fold molar excess over rGRFT (Figure 1d and Table S1). Binding of rGRFT to recombinant SARS-CoV-2 spike protein was investigated with ITC and QCM techniques. The dissociation constant (*K*_d_) of the rGRFT-spike protein complex was calculated to be 9.9 and 1.6 μM from ITC and QCM-D, respectively. ITC results provided that binding stoichiometry between rGRFT and spike is 6:1 (Figure 1e,g-left panel). Next, the binding of rGRFT to the whole virus was analyzed with an in-house developed ELISA in which heat-inactivated SARS-CoV-2 particles were coated onto wells to capture free rGRFT. In the presence of SARS-CoV-2 particles, rGRFT remained bound to wells after washing; meanwhile, statistically less rGRFT was detected onto wells in the absence of SARS-CoV-2 particles (Figure 1f). QCM-D was utilized to determine the binding affinity of rGRFT to the viral particles. rGRFT can bind heat-inactivated SARS-CoV-2 viral particles with a *K*_d_ of 4.1 μM (Figure 1g-right panel).

### rGRFT Protects Vero E6 Cells from SARS-CoV-2 Infection In Vitro

**Figure 2:**
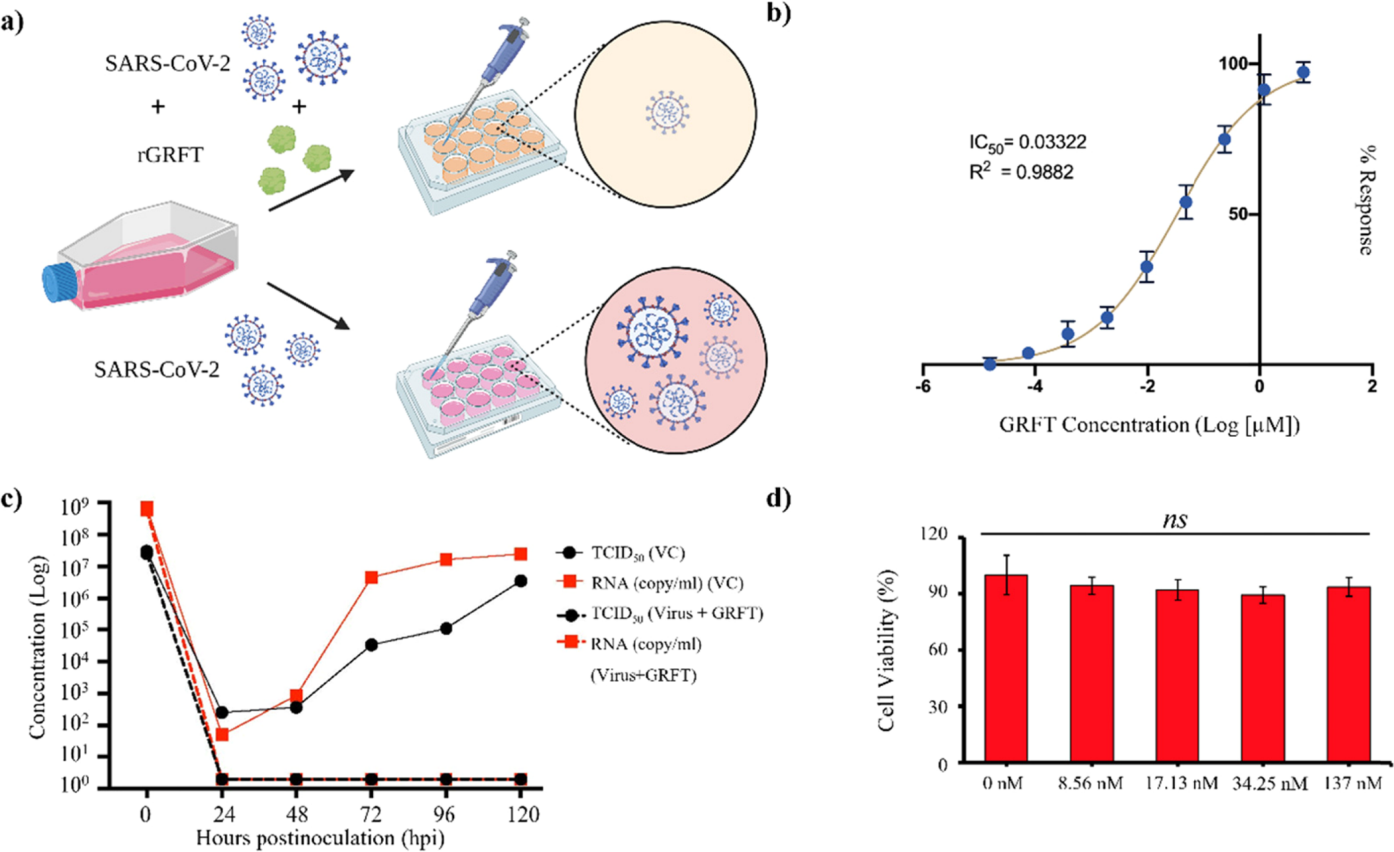
In vitro antiviral activity of rGRFT against SARS-CoV-2 infection. (a) Schematic representation of rGRFT inhibiting SARS-CoV-2 infection in Vero E6 cells, created with BioRender.com. (b) Determination of the rGRFT IC_50_ value for SARS-CoV-2 in Vero E6 cells. (c) Effects of rGRFT (5.76 nM) on SARS-CoV-2 replication in Vero E6 cells. (d) Cytotoxicity assessment of rGRFT on Vero E6 cells by MTT (3-[4,5-dimethyl-2-thiazolyl]-2,5-diphenyl-2*H*-tetrazolium bromide) assay.

The efficacy of rGRFT against SARS-CoV-2 infection was assessed in vitro by using the Vero E6 model cell line (Figure 2a). Serial diluted rGRFT solutions at concentrations ranging from 6 μM to 15.4 pM were mixed with infectious SARS-CoV-2 particles. Subsequently, cells were infected with mixtures and incubated until the cytopathic effect (CPE) reading reached 100% in the sample that did not include rGRFT. Based on the infection results, IC_50_ of rGRFT on SARS-CoV-2 infection was found to be 33.2 nM (Figure 2b). The effect of rGRFT at IC_50_ on SARS-CoV-2 replication was analyzed as well. Vero E6 cells were infected with SARS-CoV-2 in the presence and absence of rGRFT and the amount of total viral particles in the cell supernatant was determined using qPCR. rGRFT is able to suppress SARS-CoV-2 infection after at most 24 h upon inoculation (Figure 2c). In addition, rGRFT did not show any cytotoxicity effect on cells at a concentration as high as 137 nM (Figure 2d).

### rGRFT Does Not Induce Any Toxic or Immunogenic Effect to Mice

**Figure 3:**
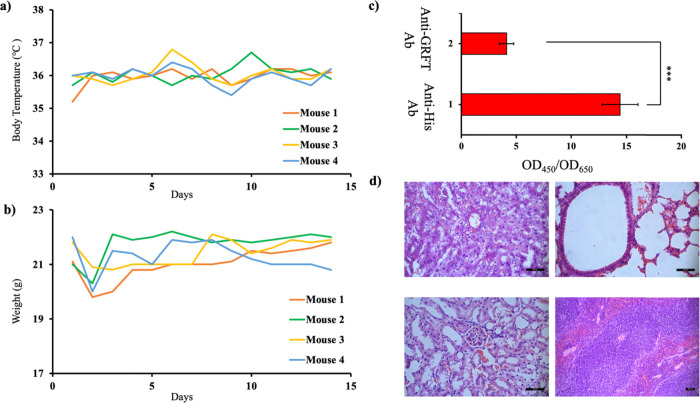
Toxicity and immunogenicity of rGRFT in vivo. (a) Body temperature and (b) weight recorded for 14 days in C57BL/6 mice during innocuity testing. (c) Immunogenicity of rGRFT administered to C57BL/6 mice during innocuity testing. Antibodies against rGRFT were significantly lower than those induced by the His-tag in the construct (*p* < 0.05). (d) Representative tissue sections of the liver (upper left), lung (upper right), kidney (lower left), and spleen (lower right) in C57BL/6 mice on the 14th day following intraperitoneal rGRFT administration (hematoxylin and eosin staining, scale bar: 50 μm).

Prior to efficacy experiments in vivo, toxicity and immunogenicity of rGRFT on live C57BL/6 mice were investigated. One hundred and fifty microliters of 100 nM rGRFT was injected into mice intraperitoneally and the mice were monitored for 14 days. rGRFT injection did not cause any meaningful variation in the body temperature and weight of mice (Figure 3a,b). During the 14 day observation period, mice did not show any evidence of local or systemic toxicity. Furthermore, biochemical parameters for the liver, kidney, and blood count results remained within the healthy range (Tables S2 and S3). Tissue samples of the liver, lung, kidney, and spleen obtained from sacrificed animals were examined under a microscope to assess histopathological effects. No visible histopathological changes were observed from the corresponding tissue samples (Figure 3d). Immunogenicity of rGRFT was analyzed with in-house developed ELISA that captures anti-rGRFT antibodies from serum via surface-immobilized rGRFT. ELISA results showed that rGRFT did not simulate the humoral immune response (Figure 3c). In addition, serum concentrations of IL-2, TNFα, and INFγ were measured with commercially available ELISA tests. No significant change in serum cytokine levels was observed between the control group and rGRFT-administered mice (Figures S1–S3).

### Intranasal Administration of rGRFT Prevents SARS-CoV-2 Infection in Mouse

**Figure 4:**
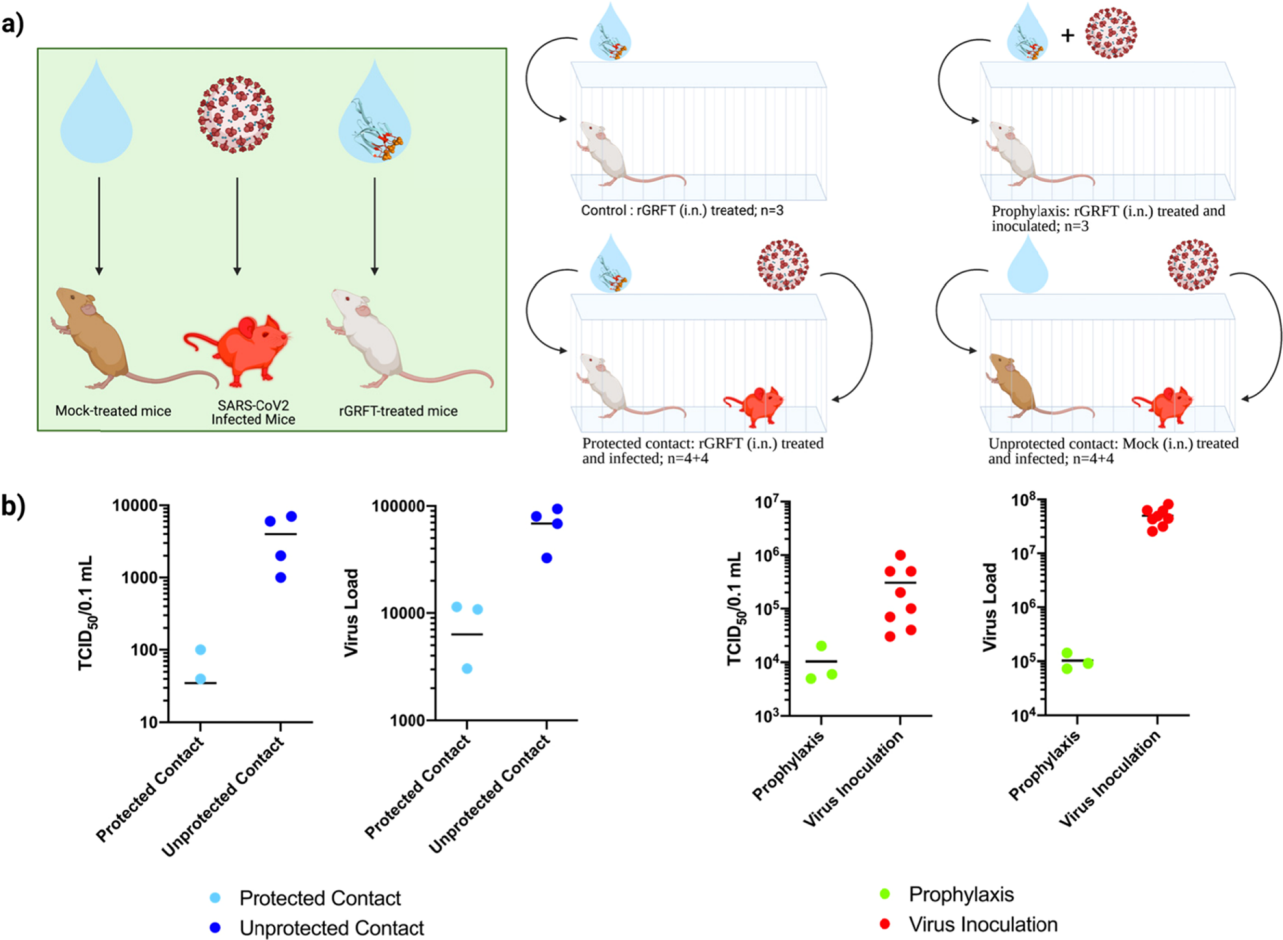
In vivo antiviral activity of rGRFT against SARS-CoV-2 infection shown for direct or contact-based infection models. (a) IFNAR^–/–^ mice assigned to various study groups. The control group is mice treated with only rGRFT (i.n.) in a cage (*n* = 3), the prophylaxis group is rGRFT (i.n.)-treated and SARS-CoV-2-inoculated mice in a cage (white mice; *n* = 3), the protected contact group is consisted of rGRFT-treated mice and SARS-CoV-2-infected mice (white mice and red mice, respectively; *n* = 4 + 4), and the unprotected contact group is mock (i.n.)-treated mice and SARS-CoV-2-infected mice (brown mice and red mice, respectively; *n* = 4 + 4). (b) SARS-CoV-2 loads and infective virus titers in lung tissues of individual IFNAR^–/–^ mice assigned to various study groups. rGRFT-treated mice from the protected contact group and mock-treated mice from the unprotected contact group are compared to SARS-CoV-2 loads and infective virus titers; the prophylaxis group (rGRFT-treated and SARS-CoV-2-infected mice) and all SARS-CoV-2-infected mouse are compared to SARS-CoV-2 loads and infective virus titers. The protected contact group values are represented with red color; the unprotected contact group values are represented with blue color; the prophylaxis group values are represented with green color; and the virus inoculation group is represented in orange color.

In vivo efficacy of rGRFT was tested in two different experimental setups. In the first setup, rGRFT was administered intranasally to mice prior to direct virus inoculation through the nose. In the second setup, mice were separated into three groups; the first mouse group was administered with rGRFT, the second mouse group remained untreated, and the third mouse group was infected intranasally with virus particles (Figure 4a). Subsequently, each member of the third mouse group was put together into a cage with either a member of the first mouse group or a member of the second mouse group. Treatments of each individual mouse were repeated for 6 days. The mice were monitored for 14 days to assess the protective role of rGRFT. In both scenarios, admission of GRFT decreases the viable infectious particle number as well as the viral load determined with qPCR (Figure 4b).

**Figure 5:**
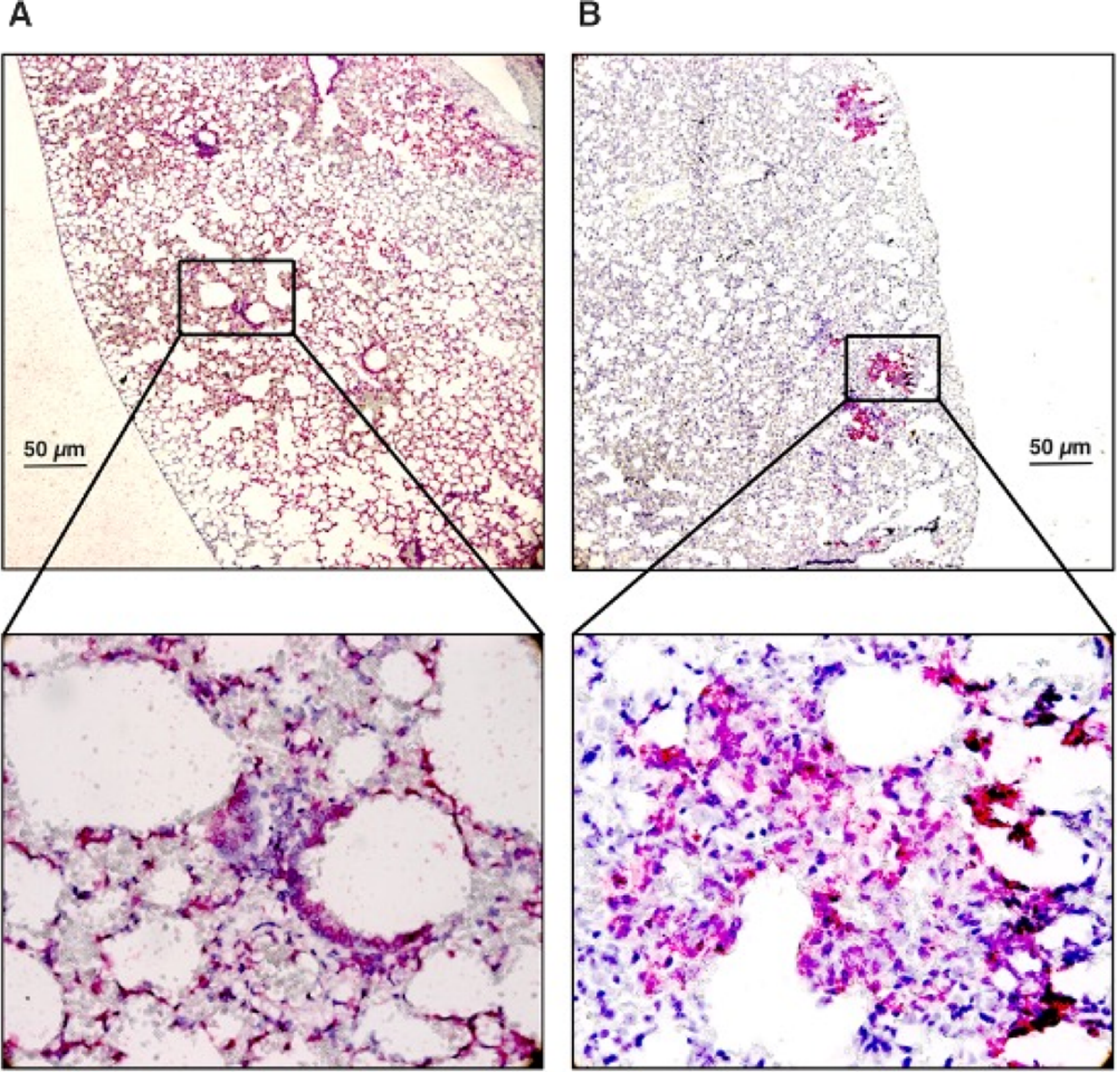
In situ hybridization of viral RNA in unprotected (A) and protected (B) animals following SARS-CoV-2 infection.

On the day of experiment termination, SARS-CoV-2 infection was monitored in lung tissues by the in situ hybridization test (ISH). The genomic RNA of the virus was detected more diffuse in the lungs of unprotected contact animals when compared to those protected; meanwhile, pattern of the infection in protected contact animals appeared patchy. SARS-CoV-2 RNA was detected in bronchial epithelial cells, alveolar epithelial cells type I and type II, and macrophages in both groups (Figure 5).

### rGRFT Can Block Transmission of SARS-CoV-2 Variants, Delta and Omicron, In Vitro

**Figure 6:**
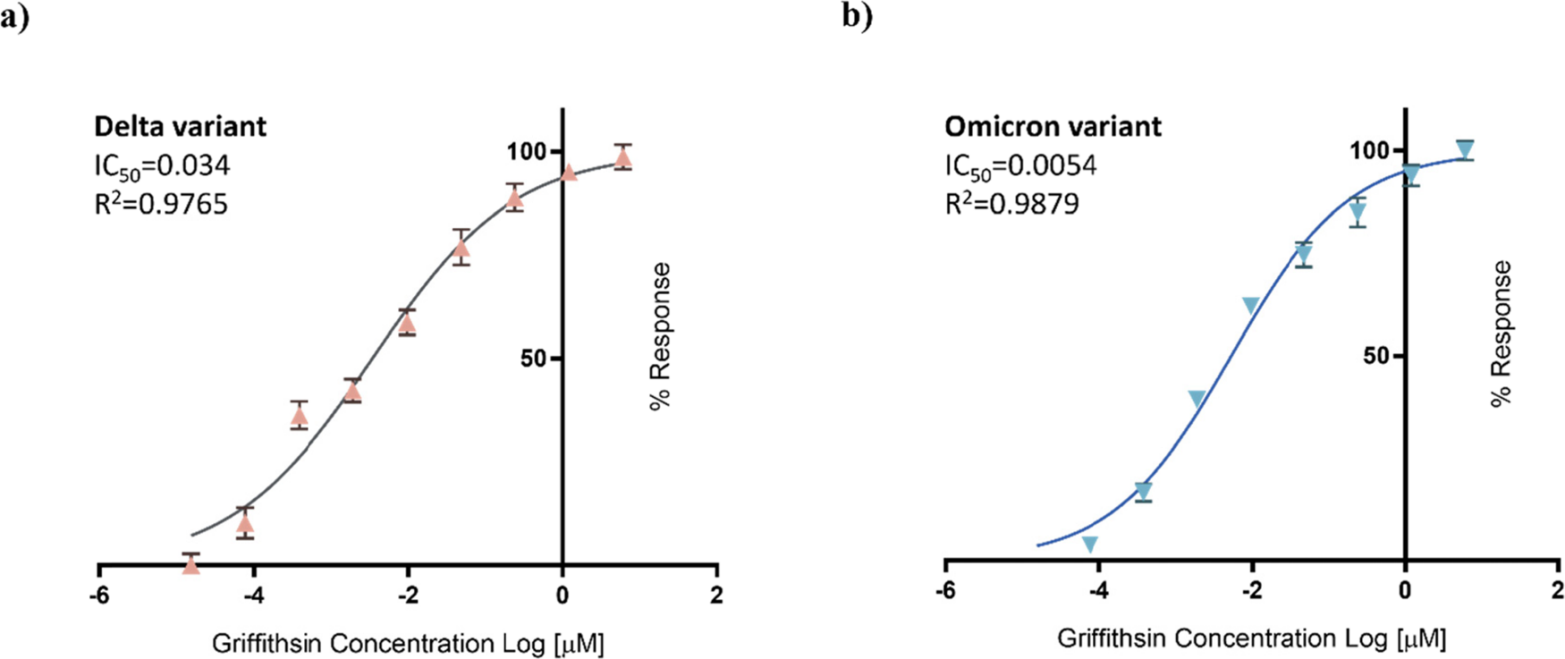
rGRFT inhibitory activity against SARS-CoV-2 variants in vitro with the Vero E6 infection model. The calculated IC_50_ value of rGRFT for Delta (a) and Omicron (b) variants.

The inhibitory effect of rGRFT was assessed for the emerged SARS-CoV-2 variants, namely, Delta and Omicron, in the in vitro Vero E6 SARS-CoV-2 infection model. To determine IC_50_ values, the infection experiment was repeated in the same process as described above. Based on the CPE readings, IC_50_ values of rGRFT against Delta and Omicron variants were found to be 34.0 and 5.4 nM, respectively. Infection results indicate that rGRFT can effectively block Delta and Omicron variants of SARS-CoV-2 (Figure 6).

## Discussion

The COVID-19 pandemic is an ongoing concern for all countries that put a significant burden on healthcare systems and the economy. Owing to the endless efforts of researchers, governments, and pharmaceutical companies, viable vaccine options against SARS-CoV-2 are available.^30^ Alternatively, neutralizing antibody therapies along with antibody mimetic peptides raise a hope to end the current pandemic in the foreseeable future.^13,31^ Yet, continuous evolution of SARS-CoV-2, especially mutations occurring in the spike protein region, pose a risk of losing or minimizing the efficacy of both vaccines and nAbs. Currently, several virus strains such as P.1 and B.1.351 can evade neutralization antibodies to a certain degree, which are determined with in vitro pseudovirus assays.^32^ Although the evolution capacity of SARS-CoV-2 S protein is unknown hitherto, low homology with other ACE2-recognizing coronaviruses such as HCoV-NL63 implied that S protein can adapt to different mutations without the loss of function.^20^ This worrisome scenario necessitates broad “pan-coronavirus” inhibitory agents to suppress the transmission until a viable vaccine is available for the emerging variants.

Here, we demonstrated that the griffithsin protein from *Griffithsia sp*. recombinantly produced in *E. coli* can bind S protein of SARS-CoV-2 in vitro and inhibit its infection in both the in vitro Vero E6 cell line and the in vivo mouse model when applied prophylactically. Toxicity assays of rGRFT with mouse models indicated that it is a tolerable agent even at concentrations higher than its therapeutic concentration window.

Previous studies showed that rGRFT can inhibit both MERS and SARS infection in in vitro cell models.^23,24^ The S protein of SARS-CoV-2 shares low-to-moderate similarity with clinically relevant MERS-CoV and SARS-CoV,^33^ yet it is proposed that the inhibitory mechanism is based on binding to glycan structures rather than amino acid sequence motifs.^23^ These glycan structures, in contrast to general amino acid sequences, are conserved between SARS-CoV and SARS-CoV-2. In addition to this, glycan sequons of SARS-CoV-2 are reported to be conserved in the course of the COVID-19 pandemic between December 2019 to April 2020.^34^ This phenomenon is correlated with another report which showed that SARS-CoV-2 mutants without glycans especially at N165 and N234 sequons have decreased affinity to ACE2 protein. In the report, the authors claimed that loss of glycans at stated sites shifts the equilibrium of RBD states to down position, which might lead to evolution of less infectious particles.^35^ Therefore, there might be a natural pressure on SARS-CoV-2 for conservation of N-glycans, which can be exploited via rGRFT treatment to hinder viral transmission.

Binding to N-linked glycans on viral receptors was proposed as a prerequisite for antiviral activity of GRFT in broad types of viruses. However, the presence of N-linked glycans in many mammalian cells raises a safety concern for use of GRFT. Contrary to other lectins such as banana lectin, concanavalin A, and cyanovirin-N (CV-N), which have unwanted side effects, GRFT is proven to be a safe microbicide in many studies involving in vitro cell culture experiments,^24,36^ in vivo experiments with macaques,^37^ and a human phase I trial.^38^ Although there is evidence for GRFT binding to human cells including peripheral blood mononuclear cells (PBMC) and human epithelial cells, its binding did not cause any mitogenic or immunogenic or toxic effect on cells.^36^ In addition, in vivo experiments on nonhuman primates and clinical trials on humans revealed that administration of GRFT through the vaginal route did not lead to any proinflammatory or toxic response.^37,38^

Aside from the efficacy and safety profile, the rGRFT production cost is reported to be as low as 3500$ per kg with a yield of approximately 2.5 g/L in *E. coli* at a scale of tons.^39^ In addition, rGRFT is reported to be stable at room temperature in PBS for 2 years without formation of aggregation and degradation byproducts.^40^ Considering the board activity of rGRFT on different coronaviruses, the evolutionary cost of glycan loss on SARS-CoV-2, the available high-yield recombinant production strategies for rGRFT, and the good stability of rGRFT at room temperature, rGRFT may provide a possible solution for the emerging variants.

## Materials and Methods

### Cloning, Expression, and Purification of Griffithsin Protein

The griffithsin (GRFT) nucleotide sequence (accession number AY744144) with N-terminal 6× His-tags and flexible GS linkers was codon-optimized for *E. coli* and chemically synthesized (Genewiz, NJ, USA) with suitable overhang sequences homologous to the pET22b cloning vector, without the pelB leader sequence. The GRFT amino acid sequence is listed in Table S4. The *grft* gene was cloned into the pet22b vector without a pelB leader sequence, using Gibson assembly. The reaction was transformed into a chemically competent strain of *E. coliDH*5α *PRO* by heat shock transformation. Selected colonies were verified by Sanger sequencing (Genewiz, USA).

For expression, the construct was transformed to the *E. coliBL*21 (*DE*3) strain. Overnight culture of cells was diluted to 1:100 in LB medium with appropriate antibiotics and was grown until the OD_600_ was 0.4–0.6. Then, the culture was induced with 1 mM IPTG for 24 h at 16 °C, 200 rpm. The cells were harvested by centrifugation and resuspended in the lysis buffer (20 mM NaH2PO4, 500 mM NaCl, 20 mM imidazole pH 7.4). The suspension was sonicated at 30% power for 10 cycles of 15 s on/45 s off. The samples were centrifuged at 21,500 *g* for 1 h and the supernatant was filtered with a 0.45 μm filter. The filtered lysate was loaded on a pre-equilibrated HisTrap nickel column (GE Life Sciences 17524701) using an ÄKTA start protein purification system. The column was washed with 10 column volumes of lysis buffer (20 mM imidazole) and 5 column volumes of lysis buffer with an imidazole gradient from 20 to 40 mM. Finally, proteins were eluted with 5 volumes of elution buffer (20 mM NaH_2_PO_4_, 500 mM NaCl, 500 mM imidazole pH 7.4). Purified griffithsin was desalted into 1× PBS (pH 7.4) using a HiTrap desalting column (GE Life Sciences) in an ÄKTA start protein purification system. Protein concentration is calculated via BCA colorimetric assay with BSA standards (Thermo Fisher Scientific).

### SDS-PAGE and Western Blotting

Samples were boiled at 95 °C for 5 min with 1× SDS loading dye and electrophoresed on 15% SDS-polyacrylamide gel. The gel was placed into Coomassie blue staining solution for ∼1 h with shaking and incubated in destaining solution (60% ddH_2_O, 30% methanol, and 10% acetic acid) until the bands were clearly visible. For the western blot, the gel was transferred to the PVDF membrane using Trans-Blot Turbo (Bio-Rad). The membrane was blocked with 5% milk in TBS-T for 1 h at room temperature. Then, the membrane was incubated in 5% milk in TBS-T containing 1:10,000 primary anti-His mouse antibodies at 4 °C, overnight. The membrane was washed in TBS-T, then incubated in 5% milk in TBS-T containing 1:10,000 horseradish peroxidase (HRP)-conjugated goat anti-mouse secondary antibodies (Abcam ab6789-1 MG) for 1 h at room temperature. After washing in TBS-T, the membrane was incubated in ECL substrates (Bio-Rad 170-5060) and visualized using Vilber Fusion Solo S.

### Circular Dichorism

A concentration of 7.5 μM rGRFT was prepared in 2 mM PBS buffer, pH = 7.4. Mannose concentrate solution was prepared with ddH_2_O and diluted in GRFT solution at 7.5, 15, 30, 60, and 120 μM final concentrations. The mannose protein mixture was incubated for 30 min at room temperature. The CD spectra of GRFT/mannose mixtures were measured from 240 to 200 nm with three repeats (Jasco J-815) at room temperature with a 300 s delay time and a 1 mm bandwidth. The secondary structure composition was calculated using the BeStSel tool developed by Micsonai et al.^41^

### ITC Assay

ITC assays were performed using MicroCal ITC200 (Malvern Panalytical). Spike protein was purchased from Synbiotik Biotechnology LLC (Ankara, Turkey); 1 μM spike protein solution and 50 μM rGRFT protein solution were prepared in 1× PBS, pH = 7.4. Two microliters of rGRFT solution was injected with 120 s intervals into the calorimetric cell containing 280 μL of spike protein solution. Buffer and dilution effects were corrected using Origin MicroCal Analysis software. Graphs were generated via GraphPad Prism software.

### QCM Assay

The QCM-D gold sensor (Biolin Scientific QSense QSX 301 Gold) surface was cleaned with piranha solution (H_2_O_2_/H_2_SO_4_ in 1:3 ratio) for 30 min at 80 °C to remove any contaminants. After piranha cleaning, the gold sensor chip was immersed in ddH_2_O for 5 min twice.

The cleaned gold sensor chip was immersed in 20 mM 11-mercaptoundecanoic acid (11-MUA) solution and incubated overnight. The gold sensor was rinsed with first 100% ethanol then ddH_2_O. The gold sensor surface coated with 11-MUA was functionalized by a QSense chamber with 400 mM EDC, which was followed by 100 mM NHS with a flow rate of 20 μL/min. The chip was rinsed with 1× PBS buffer to remove any residual EDC and NHS. Then, 150 μL of spike protein with a concentration of 400 μg/mL was introduced into the chamber with a 6.49 μL/min flow rate to make the spike protein attached to the surface. After coating the surface with spike protein, the surface was deactivated by 1 M ethanolamine HCl with a flow rate of 20 μL/min to avoid further attachment to the functionalized surface rather than spike protein. Deactivation was followed by the 1× PBS washing step to equilibrate the chamber at 20 μL/min. Afterward, 200 μL of 0.5, 1, 2, 3, 4, 5, 7.5, and 10 μM rGRFT was introduced sequentially at a 20 μL/min flow rate. Between each concentration, the chip was washed with 1× PBS at a 20 μL/min flow rate to remove any unbound molecule. First, third, fifth, seventh, and ninth overtones for frequency and dissipation values were recorded simultaneously.

The binding affinity of rGRFT toward SARS-CoV-2 virus was tested with QCM-D. The surface of the gold sensors was prepared as described above. The viral particles were attached on the surface of QCM-D via amine coupling. After the attachment of the viral particles, the activated surface as mentioned above was blocked with ethanolamine (1 M) in PBS. Final surface topographies were analyzed with atomic force microscopy as demonstrated elsewhere.^42^ Afterward, rGRFT proteins at varying concentrations of 1.7, 2.7, 5.4, 6.8, and 13.6 μM were flown on top of the inactivated and immobilized SARS-CoV-2 virus on sensor chips.

### rGRFT Binding on SARS-CoV-2 by ELISA

Ninety-six-well plates (353916, Corning) were coated with 50 μL of 10^6^ particle per mL SARS-CoV-2 isolated from Vero E6 cells by adding 150 μL of 100 mM bicarbonate/carbonate coating buffer (pH 9.6) and incubated O/N at 4 °C. The wells were washed three times with 200 μL of PBS containing 0.1% Tween-20 (Merck) (PBS-T) Two hundred microliters of blocking buffer (1% BSA in PBS-T) were added to wells and incubated for 1 h. After incubation, the blocking buffer was discarded and 100 μL of 1 μg/mL rGRFT was added to each well and incubated for 2 h. The wells are washed with 200 μL of PBS-T three times. Then, each well was incubated with a PBS-T solution containing 1:3000 primary anti-His mouse antibodies (PTGLAB 66005) for 1 h. Washing was performed with 200 μL of PBS-T. Secondary antibody solution containing 1:3000 HRP-conjugated goat anti-mouse secondary antibodies (Abcam ab6789-1 MG) in PBS-T was added to each well and incubated for 1 h. Washing steps were performed with PBS-T and wells were incubated with 100 μL of TMB substrate solution for 10 min in the dark. TMB stop solution was added and absorbance at 450 and 650 nm was measured with a microplate reader (SpectraMax M5, Molecular Devices).

### Cell and Animal Experiments

#### Virus, Cells, Animals, and Ethical Approval

SARS-CoV-2 local isolates Ank2 (Wuhan like; GenBank Acc. No: MT478019), Ank-DLT-1 (Delta variant; GenBank Acc. No: OM295705) and Ank-OmicGKS (Omicron variant) were used in the experiments requiring live virus.^42^ African green monkey kidney (Vero E6, ATCC: CRL-1586) cells, obtained from the cell culture collection of the Department of Virology, Ankara University Faculty of Veterinary Medicine, were also utilized in the study. The experiments with C57BL/6 and IFNAR^–/–^ mice were performed with permission of the Ankara University Ethical Committee for Animal Experiments (06 May 2020, 20120-8-66) in a high containment animal facility (ABSL3+), conducted according to the national regulations on the operation and procedure of animal experiments’ ethics committees (regulation no. 26220, 9 September 2006). The 14 day nonlethal SARS-CoV-2 infection model in IFNAR^–/–^ mice was previously reported.^42^

Tissue culture infective dose 50% (TCID_50_) was used to assess in vitro virus infectivity, performed as described previously.^42,43^ Quantitative genome detection was carried out by real-time reverse transcription polymerase chain reaction as reported.^42^

#### Toxicity and Immunogenicity

Vero E6 cells were grown in Dulbecco’s Modified Eagle’s Medium (DMEM) supplemented with 10% heat-inactivated fetal bovine serum, 2 mM l-glutamine, 100 units/mL penicillin, and 100 μg/mL streptomycin at 37 °C in a 5% CO_2_ humidified incubator. To determine toxicity of rGRFT on Vero E6 cells, MTT assay was used; 3 × 10^5^ cells were seeded onto 96-well plates and incubated at 37 °C in a 5% CO_2_ humidified incubator for 24 h. Then, different concentrations of rGRFT in PBS were added on cells and incubated for another 48 h. Cell viability was assessed using the MTT Cell Proliferation Assay Kit (Trevigen, 4890-25-K), according to the manufacturer’s instructions.

For innocuity testing, four C57BL/6 mice were intraperitoneally inoculated with 100 nM (250 μL) rGRFT three times with 24 h intervals and observed for 14 days with daily measurements of body temperature and weight. They were sacrificed at the end of the period with specimens for biochemical, hematological, and histopathological assessment. Mouse sera at day 14 was screened for rGRFT-specific antibodies. For this purpose, ELISA plates were coated overnight with rGRFT in bicarbonate buffer and the sera in 1/100 dilution were inoculated for an hour at room temperature. The assay was evaluated using anti-mouse-IgG-HRP conjugates and TMB substrates. The 6×-His peptide present in the recombinant protein was targeted as the control, detected using mouse anti-His antibodies, anti-mouse-IgG-HRP conjugates, and TMB substrates. The same serum samples were used to determine concentration of IFNγ, TNFα, and IL-2 using commercially available ELISA kits (BioLegend).

#### In Vitro Testing of rGRFT

In vitro virus inhibition was monitored by two approaches. Initially, neutralizing activity of rGRFT at different incubation periods was determined. For this purpose, rGRFT (6 μM) was fivefold diluted in high glucose DMEM, combined with an equal volume (100 μL) of 100 TCID_50_ of Ank2 (Wuhan like), Ank-DTL-1 (Delta variant), and Ank-OmicGKS (Omicron variant) strains of SARS-CoV-2, and the mixture was incubated at room temperature for 15 min. A 150 μL of the mixture was then inoculated on Vero E6 cells grown in a 96-well flat-bottomed tissue culture plate, with highest concentration (6 μM) of rGRFT without the virus was used as toxicity, serum-free DMEM as cell, and 75 μL of 100 TCID_50_ virus as virus controls in each plate. The plates were incubated at 37 °C in a 5% CO_2_ atmosphere and evaluated when the virus controls showed 100% CPE. The 50% inhibitory dose (IC_50_) was calculated as described elsewhere.^23^

In the second approach, Vero E6 cells grown in 24-well tissue culture plates were infected with the SARS-CoV-2 Ank2 isolate at 1 MOI and rGRFT (in IC_50_ concentration) was added at 1 h post infection. The test was incubated until virus controls showed 90% CPE. TCID_50_ values and virus genome copies were determined in culture supernatants and cell layers at termination. The test was performed in quadruplicate for all time points.

#### In Vivo Testing of rGRFT

rGRFT was delivered intranasally in 100 nmol per nostril in 50 μL volume of carrier solution including 2.5% (w/v) polyethylene glycol 1450 (PEG 1450), 0.5% benzyl alcohol, 1.5% (w/v) glycerine, 0.02% (w/v) h EDTA-diNa, and 0.02% (w/v) benzalkonium chloride. The solution was sterilized by membrane filtration (0.2 nm, Millipore, USA) and stored under chilled conditions. Intranasal virus inoculations were carried out as 10^3^TCID_50_ in 50 μL per nostril.

A total of 28 IFNAR^–/–^ mice (8–12 weeks) were randomly divided into six groups for prophylaxis and contact experiments as well as control individuals in the prophylaxis group received intranasal rGRFT and were inoculated with SARS-CoV-2 Ank2 after 4 h. Each animal further received the same amount and route of rGRFT following 4 subsequent days. The protective effect of rGRFT was tested with a contact scenario in pairs housed in the same cage. One individual in each cage was intranasally inoculated with the virus and the accompanying mouse received rGRFT (protected contact) or null carrier solution (unprotected contact) (Figure 2). The rGRFT and carrier treatments were repeated at the same time for 6 additional days. Animals were monitored clinically for 14 days with tail vein blood specimens collected on days 0 and 7. At the end of the period, the animals were humanely euthanized and tissue specimens were collected for histopathological evaluation and immunohistochemistry, performed as previously described.^42^

## Supporting Information Available

Serum TNFα concentration of rGRFT-administered mice compared with the control group (Figure S1); serum INFγ concentration of rGRFT-administered mice compared with the control group (Figure S2); serum IL-2 concentration of rGRFT-administered mice compared with the control group (Figure S3); calculated 2D structure of rGRFT and its complexes with mannose at the molar ratio (Table S1); blood biochemistry results for main indicators of liver and kidney functions in C57BL/6 mice on the 14th day following intraperitoneal rGRFT administration (Table S2); hematologic parameters in C57BL/6 mice on the 14th day following intraperitoneal rGRFT administration (Table S3); and amino acid sequence of rGRFT (Table S4) (PDF)

## Notes

All of the raw data is available from lead author upon request.
