# Supplementary material for: A Highly Potent SARS-CoV-2 Blocking Lectin Protein
Source: ACS Infect Dis. 2022 Apr 15;8(7):1253–64. doi: 10.1021/acsinfecdis.2c00006 (PMC9017247; doi:10.1021/acsinfecdis.2c00006)
Supplement: Supplementary file 1 — id2c00006_si_001.pdf [file id2c00006_si_001.pdf]

Supporting Information

A Highly Potent SARS-CoV-2 Blocking Lectin Protein

Recep E. Ahan[1,†], Alireza Hanifehnezhad[2,†], Ebru Ş. Kehribar[1,†], Tuba C. Oguzoglu[2], Katalin Földes[2], Cemile E. Özçelik[1], Nazlican Filazi[2], Sıdıka Öztop[3], Fahreddin Palaz[4], Sevgen Önder[5], Eray U. Bozkurt[1], Koray Ergünay[6*], Aykut Özkul[2,7,*], Urartu Ö. Ş. Şeker[1,*]

[1]UNAM-Institute of Materials Science and Nanotechnology, Bilkent University; Ankara 06800, Turkey.

[2]Faculty of Veterinary Medicine, Department of Virology, Ankara University; Ankara 06110, Turkey.

[3]Adana Dr. Turgut Noyan Medical and Research Center, Department of Immunology, Baskent University; Adana 01250, Turkey.

[4]Faculty of Medicine, Undergraduate Medical Education Program, Hacettepe University; Ankara 06230, Turkey

[5]Faculty of Medicine, Department of Medical Pathology, Hacettepe University; Ankara 06230, Turkey.

[6]Faculty of Medicine, Department of Medical Microbiology, Virology Unit, Hacettepe University; Ankara 06230, Turkey.

[7]Biotechnology Institute, Ankara University; Ankara 06135, Turkey.

†*These authors have contributed equally*

*Corresponding authors

aykut.ozkul@ankara.edu.tr , korayergunay@gmail.com , urartu@bilkent.edu.tr

Figure S1. Serum TNFα concentration of rGRFT administered mice compared with control group.

Figure S2. Serum INFγ concentration of rGRFT administered mice compared with control group.

Figure S3. Serum IL-2 concentration of rGRFT administered mice compared with control group.

Table S1. Calculated 2D structure of rGRFT and its complexes with mannose at molar ratio.

Table S2. Blood biochemistry results for main indicators of liver and kidney functions in C57BL/6 mice on the 14th day following intraperitoneal rGRFT administration.

Table S3. Hematologic parameters in C57BL/6 mice on the 14th day following intraperitoneal rGRFT administration

Table S4. Amino acid sequence of rGRFT

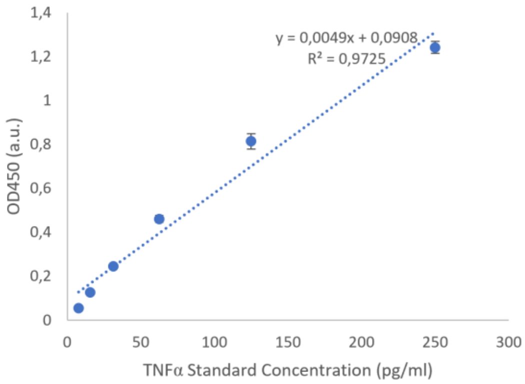

Sample Name	Absorption	Calculated concentration*
rGRFT mouse 1	-0,0021	ND
rGRFT mouse 2	0,0005	ND
rGRFT mouse 3	0,0503	ND
rGRFT mouse 4	0,0004	ND
Control 1	-0,001	ND
Control 2	0,0024	ND
Control 3	0,0192	ND

Figure S1. Serum TNFα concentration of rGRFT administered mice compared with control group. *If calculated concentration is below zero, "not determined (ND)" is written instead of calculated negative concentration.

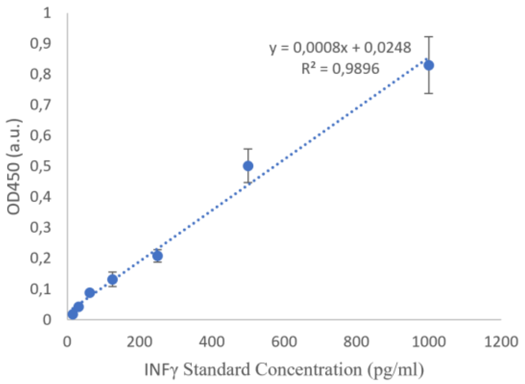

Sample Name	Absorption	Calculated concentration*
rGRFT mouse 1	0,0048	ND
rGRFT mouse 2	0,0077	ND
rGRFT mouse 3	0,0365	ND
rGRFT mouse 4	0,0087	ND
Control 1	-0,0020	ND
Control 2	0,0024	ND
Control 3	0,02855	ND

Figure S2. Serum INFγ concentration of rGRFT administered mice compared with control group. *If calculated concentration is below zero, "not determined (ND)" is written instead of calculated negative concentration.

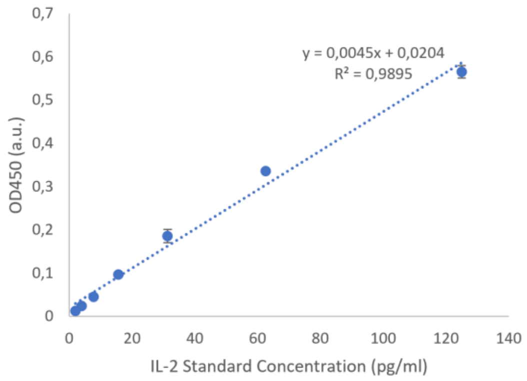

Sample Name	Absorption	Calculated concentration*
rGRFT mouse 1	-0,0002	ND
rGRFT mouse 2	0	ND
rGRFT mouse 3	0,0002	ND
rGRFT mouse 4	0,0015	ND
Control 1	0,0043	ND
Control 2	0,006	ND
Control 3	-0,0008	ND

Figure S3. Serum IL-2 concentration of rGRFT administered mice compared with control group. *If calculated concentration is below zero, "not determined (ND)" is written instead of calculated negative concentration.

Table S1. Calculated 2D structure of rGRFT and its complexes with mannose at molar ratio.

	GRFT	GRFT + Mannose (1:1)	GRFT + Mannose (1:2)	GRFT + Mannose (1:4)	GRFT + Mannose (1:8)	GRFT + Mannose (1:16)
α-Helix	3.1%	2.6%	0.0%	0.0%	0.5%	0.0%
ß-sheet (Antiparallel)	42.2%	37.6%	41.5%	43.6%	40.8%	42.4%
ß-sheet (Parallel)	3.1%	10.3%	5.2%	6.7%	6.8%	0.2%
Turns	10.5%	10.8%	9.5%	7.0%	9.3%	10.3%
Others	41.0%	38.8%	43.9%	42.7%	42.7%	47.1%

Table S2. Blood biochemistry results for main indicators of liver and kidney functions in C57BL/6 mice on the 14th day following intraperitoneal rGRFT administration, n=4. (ALB: albumin, ALT: alanine aminotransferase, ALP: alkaline phosphatase, AST: aspartate aminotransferase, BUN: blood urea nitrogen, CRE: creatinine, GLU: glucose).

Parameters							
	ALB (g/dL)	ALT (U/L)	ALP (U/L)	AST (U/L)	BUN (mg/dL)	CRE (mg/dL)	GLU (mg/dL)
Average	2,95 ± 0,29	66,5 ± 6,56	126,75 ±14,39	133,25 ±17,69	16,75 ±3,40	0,275 ±0,05	202,75 ±27,46
Range	2,6 - 3,3	58 - 73	109-144	116-158	12 - 20	0,2 – 0,3	174-240

Table S3. Hematologic parameters in C57BL/6 mice on the 14th day following intraperitoneal rGRFT administration, n=4. (WBC; white blood cells, RBC: erythrocytes, HGB: hemoglobin, PLT: platelets, NEUT: neutrophiles, LYMP: lymphocytes, MON: monocytes, EOS: eosinophiles, BASO: basophiles, HCT: hematocrit). For EOS, one mouse was excluded as outlier (0,71).

Parameters										
	WBC (K/µL)	RBC (M/µL)	HGB (g/dL)	PLT (K/µL)	NEUT (K/µL)	LYMP (K/µL)	MON (K/µL)	EOS (K/µL)	BASO (K/µL)	HCT (%)
Averg.	7,52 ±2,68	9,29 ±1,88	12,8 ±3,07	857 ±257,08	1,68 ±0,94	8,39 ±3,82	0,75 ±0,33	0,07 ±0,05	0,04 ±0,06	53,5 ±9,71
Range	4,57-10,9	7,57-11,2	9,78-16,3	568-1179	0,94-3,04	3,51-12,3	0,38-1,01	0,01-0,12	0-0,12	41-63

Table S4. Amino acid sequence of rGRFT

rGRFT amino acid sequence	MGSSHHHHHHSSGLVPRGSLTHRKFGGSGGSPFSGLSSIAVRSGSYLDAIII DGVHHGGSGGNLSPTFTFGSGEYISNMTIRSGDYIDNISFETNMGRRFGPYG GSGGSANTLSNVKVIQINGSAGDYLDSLDIYYEQY**
